# Potential effectiveness of parenteral nemonoxacin in the treatment of *Clostridioides difficile* infections: *in vitro*, *ex vivo*, and mouse studies

**DOI:** 10.3389/fmicb.2024.1418817

**Published:** 2024-08-20

**Authors:** Ching-Chi Lee, Xiang-Zhe Yan, Hung-Tsung Wu, Wen-Chien Ko, Pei-Jane Tsai, Yuan-Pin Hung

**Affiliations:** ^1^Clinical Medicine Research Center, National Cheng Kung University Hospital, College of Medicine, National Cheng Kung University, Tainan, Taiwan; ^2^Department of Internal Medicine, National Cheng Kung University Hospital, College of Medicine, National Cheng Kung University, Tainan, Taiwan; ^3^Department of Microbiology and Immunology, College of Medicine, National Cheng Kung University, Tainan, Taiwan; ^4^Department of Medicine, College of Medicine, National Cheng Kung University, Tainan, Taiwan; ^5^Institute of Basic Medical Sciences, College of Medicine, National Cheng Kung University, Tainan, Taiwan; ^6^Department of Medical Laboratory Science and Biotechnology, College of Medicine, National Cheng Kung University, Tainan, Taiwan; ^7^Department of Pathology, National Cheng Kung University Hospital, College of Medicine, National Cheng Kung University, Tainan, Taiwan; ^8^Department of Internal Medicine, Tainan Hospital, Ministry of Health and Welfare, Tainan, Taiwan

**Keywords:** nemonoxacin, *Clostridium difficile* infection, mouse model, parenteral administration, fluoroquinolone

## Abstract

**Introduction:**

Antimicrobial therapy plays a crucial role in the management of CDI patients. However, the standard agent for treating CDIs is limited to oral fidaxomicin or vancomycin. For patients made nil by mouth, there is a clinically urgent and essential need to develop an intravenous antibiotic.

**Methods:**

For *C. difficile* with the lowest MIC of nemonoxacin and vancomycin, the inhibitory effects were tested using the kinetic time-kill assay and ex vivo co-culture model. The effectiveness of nemonoxacin and vancomycin in inhibiting spore germination, the sporicidal activity, and the treatment of mice with CDIs were compared.

**Results:**

For clinical isolates and laboratory strains, lower MICs of nemonoxacin against *C. difficile* than levofloxacin and ciprofloxacin were observed, even in those harboring point mutations in the quinolone-resistance determining region. Although nemonoxacin failed to suppress spore outgrowth and germination in *C. difficile*, it exhibited an effective inhibitory effect against *C. difficile* in the kinetic time-kill assay and the *ex vivo* co-culture model. Mice receiving intraperitoneal nemonoxacin had less weight loss, higher cecum weight, a longer colon length, and lower expression of the *tcdB* gene, compared with untreated mice. Notably, there were no significant differences observed in weight loss, cecum weight, colon length, or tcdB gene expression between mice treated with vancomycin and those treated with any dose of nemonoxacin. Similarly, no significant differences were found between mice receiving combination therapy of intraperitoneal nemonoxacin plus oral vancomycin and those treated with intraperitoneal nemonoxacin or oral vancomycin alone.

**Discussion:**

The potential role of nemonoxacin, which can be administered parenterally, for treating CDIs was evidenced through the *in vitro*, *ex vivo*, and mouse models.

## Introduction

*Clostridioides difficile*, a gram-positive, obligate anaerobic bacterium, is the most common etiology of antibiotic-associated nosocomial diarrhea and has been traditionally identified as the etiologic microorganism of pseudomembranous colitis (Hall and O’toole, 1935; George et al., 1978). With an increasing worldwide incidence, *C. difficile* infections (CDIs) substantially quadruple the expenditure of hospitalization (Leffler and Lamont, 2015), and antimicrobial therapy currently plays a crucial role in terminating the pathogenesis of CDIs (Leffler and Lamont, 2015). According to the practice guideline updated by the Infectious Diseases Society of America (IDSA) (McDonald et al., 2018), oral administration of fidaxomicin or vancomycin is the standard in treating adults experiencing initial or recurrent episodes of CDIs. However, approximately 3 to 8% of CDI patients develop a fulminant disease, characterized by complications such as perforation, severe ileus with toxic megacolon, and progression to hypotension or septicemia (Khan and Elzouki, 2014). Therefore, parenteral administration of effective antimicrobials is essential for such patients who need to be nil by mouth (NPO). Furthermore, parenteral metronidazole has been recognized as an alternative treatment option for patients with mild to moderate CDIs (Bauer et al., 2009; Shannon-Lowe et al., 2010). However, a previous study had reported a relatively poor response to parenteral metronidazole (Musher et al., 2005), and it has shown a slower treatment response compared with oral vancomycin therapy (Wilcox and Howe, 1995). Therefore, the development of a novel and effective anti-*C. difficile* agent that can be administered parenterally is urgent and crucial.

Nemonoxacin, developed by TaiGen Biotechnology Co., Ltd. (Taipei, Taiwan), is an oral and intravenous quinolone that features a C-8-methoxy nonfluorinated structure (Li et al., 2010). Nemonoxacin exhibits potent activity against Gram-positive, Gram-negative, and atypical pathogens (Li et al., 2010), and it can safely and effectively treat community-acquired pneumonia, skin and soft-tissue infections, and diabetic foot infections (Poole, 2014). Importantly, numerous investigations have demonstrated that nemonoxacin has lower minimum inhibitory concentrations (MICs) against clinical *C. difficile* isolates than commercially available fluoroquinolones, such as levofloxacin, ciprofloxacin, and moxifloxacin (Lin et al., 2011; Liao et al., 2012; Huang et al., 2015). Moreover, similar MIC ranges for nemonoxacin and vancomycin or metronidazole were observed in *C. difficile* clinical isolates in Taiwan (Lin et al., 2011, Liao et al., 2012). Therefore, we hypothesized that the unique properties of this non-fluorinated quinolone make it a potential therapeutic agent for parenterally treating patients with CDIs. In the present study, the effectiveness of nemonoxacin in treating CDIs was examined through *in vitro*, *ex vivo*, and mouse studies.

## Methods

### *Clostridioides difficile* isolate/strain

Based on a previous report detailing a nationwide surveillance in Taiwan (Hung et al., 2018), eight clinical *C. difficile* isolates, namely 104-NCKU I2, 104-CSMU B42, 104-CMMC 42, 104-TNHP 235, 105-NTUH 34, 105-NTUH 50, 105-NTUH 61, and 105-NCKU 114, were tested. Four laboratory strains previously reported, in terms of VPI 10463 (Chiu et al., 2021), ATCC 700057 (Darkoh et al., 2011), ATCC BAA-1805 (Hung et al., 2016), and Netherlands RT078 (Hung et al., 2016), were also studied.

### Antimicrobial susceptibility and quinolone-resistance determining region (QRDR)

Overnight cultures of the *C. difficile* isolate/strain were inoculated onto brucella broth supplemented with brucella broth (BD Life Science, 28 mg/mL), hemin (Sigma-Aldrich, 5 mg/L), vitamin K1 (Sigma-Aldrich, 0.5 mg/L), and 5% lysed horse blood (Creative Life Sciences Co., Ltd.). The MICs of the tested antibiotics, such as nemonoxacin, levofloxacin, moxifloxacin, and vancomycin, were assessed using the method of broth microdilution recommended by the Clinical Laboratory and Standards Institute in 2023 (CLSI, 2023).

Sequence analysis of *gyrA* and *gyrB* in the QRDR was performed based on a previously established method (Dridi et al., 2002). In brief, the DNA region was subjected to amplification employing primer pairs: *gyrA1*- *gyrA2* for *gyrA* and *gyrB1*- *gyrB2* for *gyrB*. Products recognized by polymerase chain reaction (PCR) were purified and sequenced. Finally, pairwise alignments of the DNA sequences were conducted utilizing the BLAST server of the National Center for Biotechnology.

### Kinetic time-kill assay

*Clostridioides difficile* isolate/strain was grown anaerobically overnight at 37°C in BHIS (brain heart infusion supplemented with l-cysteine [0.1%, Sigma, United Kingdom] and yeast extract [5 mg/mL, Oxoid]) broth. The bacterial suspension was adjusted to McFarland 0.5 in sterile water, then diluted 100-fold with BHIS broth, and 2 mL was added to a test tube containing different concentrations of the tested antibiotics, resulting in a final volume of 4 mL. To identify the optimal dosages of nemonoxacin, we tested 0.5×, 1×, and 4× MICs of nemonoxacin, and compared the results with the 1× MIC of vancomycin. After 0, 4, 8, 12, 24, and 48 h, 100 μL of the bacterial culture was removed and centrifuged at 4000 *g* for 10 min. The supernatant was discarded and bacteria were resuspended in 100 μL sterile water. After serial 10-fold dilutions, a volume of 30 μL from each dilution was cultured on CDC anaerobic blood agar plates for calculating the colony-forming unit (CFU).

### Spore germination and sporicidal assay

After preparation of *C. difficile* spores as previously described (Yang et al., 2018), the effect of the tested antibiotics on spore germination and sporicidal activity was examined. Briefly, five microliters of the spore stock solution were dissolved in 2 mL of BHIS broth, heated at 60°C for 30 min, and then adjusted to an OD_600_ value of 0.2 with BHIS broth. The 200 μL of spore suspension was treated with 200 μL of tested antibiotics at different concentrations at room temperature for 30 min and then antibiotics were removed by centrifugation. To recognize the optimal dosages of nemonoxacin, we tested 0.5×, 1×, and 4× MICs of nemonoxacin, and compared the results with the 1× MIC of vancomycin. Ninety microliters of spores were transferred to a 96-well cell culture plate and added with 10 μL of 100 mM taurocholate acid (TA, Sigma-Aldrich). Spore germination was measured with OD_600_ spectrophotometrically for 12 min with 1-min intervals. The quantification results are shown as the ratio of OD_600_ at time X to OD_600_ at time zero.

The sporicidal activity of the tested antibiotics was measured after triggering spore germination by TA. To identify the optimal dosages of nemonoxacin, we tested 0.5×, 1×, and 4× MICs of nemonoxacin, and compared the results with the 1× MIC of vancomycin. Fifty microliters of serially diluted samples were plated onto CDC anaerobic blood agar plates at 37°C in the anaerobic chamber for 48 h, and the CFU of each diluted sample was measured.

### *Ex vivo* co-culture assay

As previously described (Horvat and Rupnik, 2018), a laboratory *C. difficile* strain was anaerobically cultured on CDC anaerobic blood agar plates at 37°C for 48 h and then adjusted to McFarland 0.5 in PBS. The stool was collected from six 7-week-old male mice that had received the antibiotic cocktail for 4 days and dissolved in PBS. Forty microliters of stool were added to an Eppendorf, inoculated with 10^6^ CFU of *C. difficile*, and treated with antibiotics to reach a final volume of 160 μL. To identify the optimal dosages of nemonoxacin, we tested 0.5×, 1×, and 4× MICs of nemonoxacin, and compared the results with the 1× MIC of vancomycin. The cultures were grown anaerobically for 24 h at 37°C, and 50 μL of samples were serially diluted with sterile water. These dilutions were then incubated anaerobically on cycloserine-cefoxitin fructose agar (CCFA) plates with egg yolk enrichment agar plates (Dybo Enterprise Company Limited) at 37°C for 48 h to calculate the CFU.

### CDI mouse model

As the previously established model (Hung et al., 2015), wild-type C57BL/6JNarl male mice were obtained from the National Laboratory Animal Center in Tainan. Seven to eight-week-old (20–25 g) B6 mice were given a daily administration of an antibiotic mixture (vancomycin, 0.045 mg/mL; metronidazole, 0.215 mg/mL; kanamycin, 0.4 mg/mL; colistin, 0.057 mg/mL; and gentamycin, 0.035 mg/mL) in their drinking water for a duration of 5 days before oral inoculation of *C. difficile* spores. Metronidazole and vancomycin were not administered during one-day period before the inoculation to prevent interference with the *C. difficile* colonization. On the day of oral inoculation, the mice received clindamycin at a dosage of 4 mg/kg via intraperitoneal injection, esomeprazole (Nexium^®^, 0.1 mg) by oral gavage, followed by oral feeding with *C. difficile* spores (10^6^ CFU). Mice were weighed and symptoms of CDI were recorded daily, until they were sacrificed at 48 h post-infection. Mice were bred and housed in the animal facility of National Cheng Kung University. All animal studies were performed according to the protocols approved by the Institutional Animal Care and Use Committee of National Cheng Kung University.

#### Protocol of nemonoxacin and vancomycin treatment in mice

The mice were treated with tested antibiotics at specific time points (8, 24, 32, and 48 h) and sacrificed at 52 h after oral inoculation. To identify the optimal dosages of nemonoxacin monotherapy in mice, mice were studied in each dosage group of 10, 20, 40, or 60 mg/kg of nemonoxacin, which were compared with 25 mg/kg of vancomycin. Next mice receiving 20 mg/kg of nemonoxacin plus 25 mg/kg of vancomycin was compared with those of the uninfected group, the no-antibiotic group, the group receiving 20 mg/kg of nemonoxacin, and the group receiving 25 mg/kg of vancomycin. The mice number was at least five in each experiment group and was repeated for four times. The efficacy of the tested antibiotics was assessed by the survival day, body weight, cecum weight, colon length, and the relative expression of the *tcdB* gene in mouse stool. All data presented are representative of four independent experiments.

### Detection of toxin gene

After sacrificing the mice, the stool in the colon were preserved in 500 μL of DNA/RNA Shield (Pangea Laboratory) at −20°C. Subsequently, the stool samples were homogenized using a MagNA Lyser Instrument (Roche Applied Science), and the DNA was extracted from the stool using the High Pure PCR Template Preparation Kit (Roche, Mannheim, Germany). As previously described (Houser et al., 2010), the extracted DNA was amplified for detecting the *tcdB* gene of *C. difficile* using real-time PCR.

### Statistical analysis

Statistical analysis was performed using GraphPad Prism software version 9. Continuous variables were expressed as the mean ± standard deviation. Continuous variables between the different antibiotic groups were compared using the Mann–Whitney *U* test in the *ex vivo* co-culture assay and mouse model. Statistical significance between groups was defined as a two-tailed *p*-value less than 0.05.

## Results

### MIC of fluoroquinolone and mutation in QRDR

The MICs of nemonoxacin, levofloxacin, and moxifloxacin against eight clinical isolates and four laboratory strains were detailed in Table 1. The MIC_50_ for nemonoxacin, levofloxacin, moxifloxacin, and vancomycin were 1, 2, 1, and 0.25 μg/mL, respectively; and the MIC_90_ for these tested antibiotics were 8, >32, 16, and 0.5 μg/mL, respectively (Supplementary Figure 1). Of four clinical isolates (104-CMMC 42, 104-TNHP 235, 105-NTUH 61, and 105 NCKU 114) and one laboratory strain (ATCC BAA-1805) with a nemonoxacin MIC ≥4 μg/mL, point mutations (Thr82Ile) in *GyrA* or (Asp426Asn) in *GyrB* were disclosed (Table 1). Notably, for these *C. difficile* isolates/strains harboring points mutations in QRDRs, the MIC of nemonoxacin was lower than those of levofloxacin and moxifloxacin.

**Table 1:** Antibiotic susceptibilities and point mutation in the quinolone-resistance determining region for 8 clinical isolates and 4 laboratory strains.

	Ribotype (RT)	Nemonoxacin MIC (μg/mL)	Levofloxacin MIC (μg/mL)	Moxifloxacin MIC (μg/mL)	Vancomycin MIC (μg/mL)	*GyrA* mutation	*GyrB* mutation
Clinical isolates							
104-NCKU 12	RT002	0.5	2	1	0.25	ND	ND
104-CSMU B42	ND	1	2	1	0.25	ND	ND
104-CMMC 42	RT078	4	>32	16	0.5	Thr82Ile	ND
104-TNHP 235	RT017	8	>32	16	0.25	Thr82Ile	ND
105-NTUH 50	RT027	0.5	2	1	0.25	ND	ND
105-NTUH 34	RT126	0.5	2	1	0.5	ND	ND
105-NTUH 61	RT078	8	>32	4	0.25	ND	Asp426Asn
105-NCKU 114	ND	4	>32	16	0.25	Thr82Ile	ND
Laboratory strains							
ATCC BAA-1805	RT027	8	>32	16	0.25	Thr82Ile	ND
ATCC 700057	RT038	1	1	1	0.5	ND	ND
VPI 10463	RT087	0.5	1	0.5	0.5	ND	ND
Netherland RT078	RT078	0.5	1	0.5	0.5	ND	ND

ND, not detected.

Of the total 12 *C. difficile* presented in Table 1, one (i.e., VPI 10463) with the lowest MIC of nemonoxacin and vancomycin among eight clinical isolates and/or another (i.e., 105-NTUH 50) with the lowest MIC of nemonoxacin and vancomycin in four laboratory strains were chosen for further kinetic inhibitory effects, *ex vivo* models, and animal studies.

### Kinetic inhibitory effect

**Figure 1:**
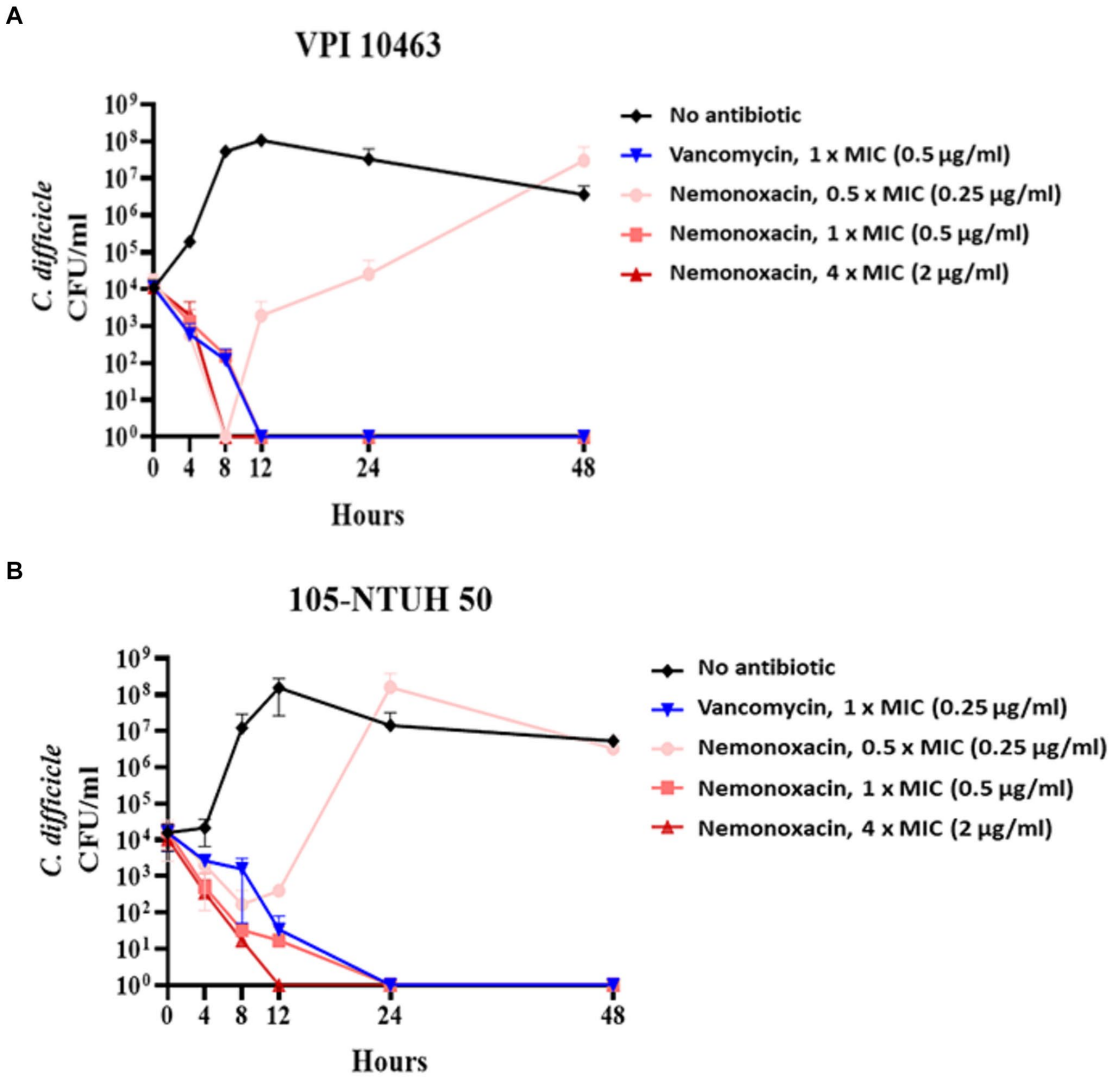
The kinetic inhibitory effect of various concentrations (0.5×, 1×, and 4× MIC) of nemonoxacin and 1× MIC of vancomycin on one clinical isolate (VPI 10463, **A**) and one laboratory strain (105-NTUH 50, **B**). CFU, Colony-forming unit; MIC, minimum inhibitory concentrations.

For VPI 10463 (Figure 1A) and 105-NTUH 50 (Figure 1B), similar to vancomycin, inhibitory effects were observed at 1 × MICs of nemonoxacin. Additionally, faster inhibitory effects were noted at 4 × MICs of nemonoxacin compared with vancomycin.

### Spore germination and sporicidal effect

**Figure 2:**
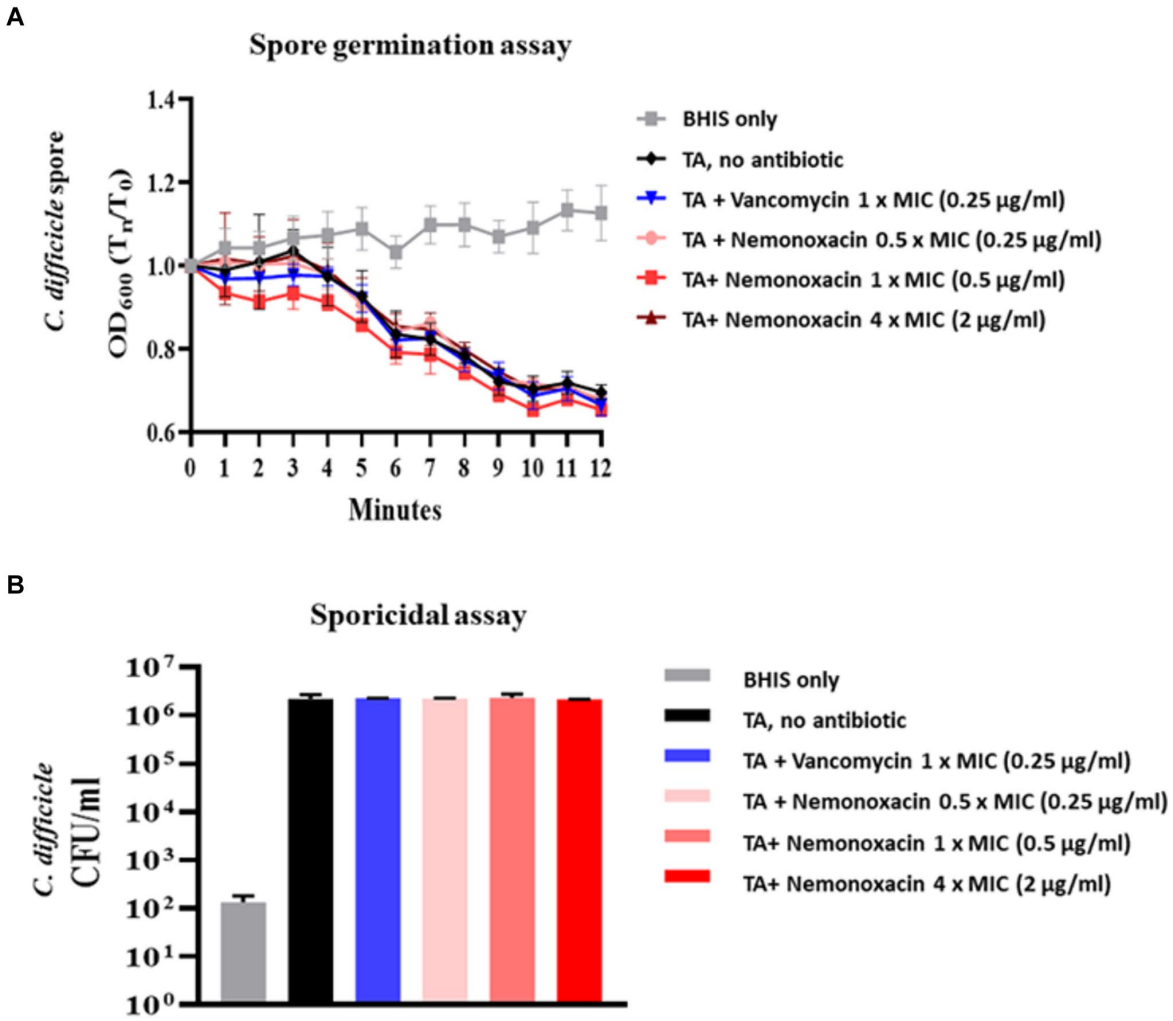
Effects of various concentrations (0.5×, 1×, and 4× MIC) of nemonoxacin and 1× MIC of vancomycin on spore germination (Figure 2A) and sporicidal activity (Figure 2B) in one clinical isolate (105-NTUH 50)^#^. BHIS, Brain-heart infusion; CFU, colony-forming unit; MIC, minimum inhibitory concentrations; TA, taurocholate acid. ^#^ OD_600_ indicated the optical density at 600 nm and variables were compared between indicated groups using independent *t*-test.

Consistent with vancomycin, there were no significant changes in OD600 values in the groups with no treatment and any concentration of nemonoxacin for purified spores from 105-NTUH 50. This indicates that nemonoxacin could not inhibit the germination of *C. difficile* spores (Figure 2A). Furthermore, consistent with vancomycin, nemonoxacin failed to inhibit the growth of *C. difficile* spores in the sporicidal assay (Figure 2B).

### *Ex vivo* co-culture model

**Figure 3:**
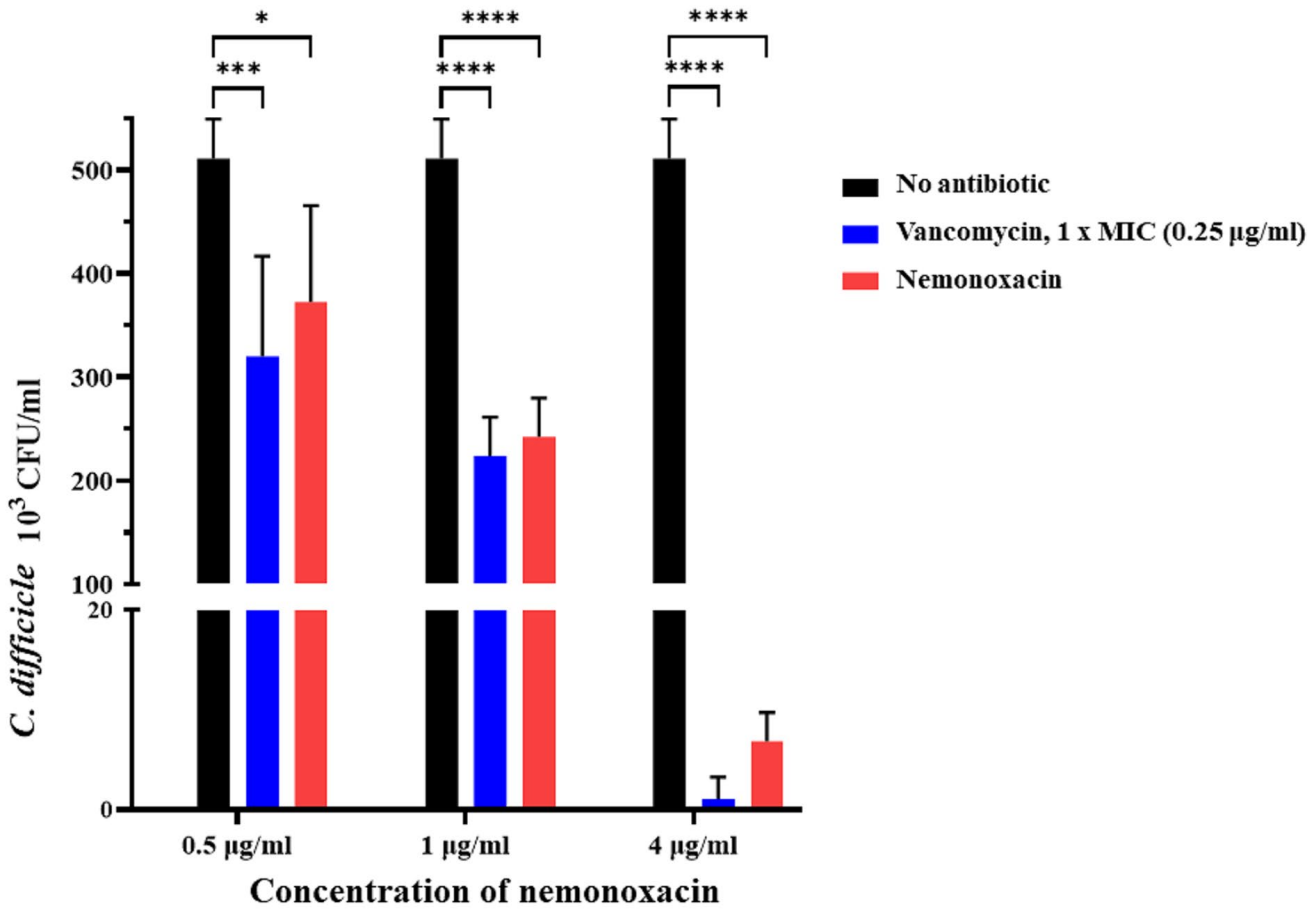
*Ex vivo* effects of various concentrations (0.5×, 1×, and 4× MIC) of nemonoxacin and 1× MIC of vancomycin on inhibiting one laboratory strain (VPI 10463) in a co-culture model. The stool was collected from six 7-week-old male mice that had received the antibiotic cocktail for 4 days. Values are expressed as the mean ± standard deviation (*p < 0.05; **p < 0.01; ***p < 0.001; ***p < 0.0001 relative to control group). All data are representative of three independent experiments. MIC, minimum inhibitory concentrations.

In the co-culture model with VPI 10463, when compared with the CFU count of *C. difficile* in the untreated group (CDI group, Figure 3), the count significantly decreased by 27% (*p* = 0.017), 52.6% (*p* < 0.001), and 98.7% (*p* < 0.001) in the nemonoxacin groups with concentrations of 0.5, 1, and 4 μg/mL, respectively. Furthermore, the CFU count of *C. difficile* showed a significant difference between the nemonoxacin groups of 0.5 and 1 μg/mL (*p* = 0.037) as well as between the nemonoxacin groups of 1 and 4 μg/mL (*p* < 0.001).

### Intraperitoneal nemonoxacin vs. oral vancomycin in the mouse model

**Figure 4:**
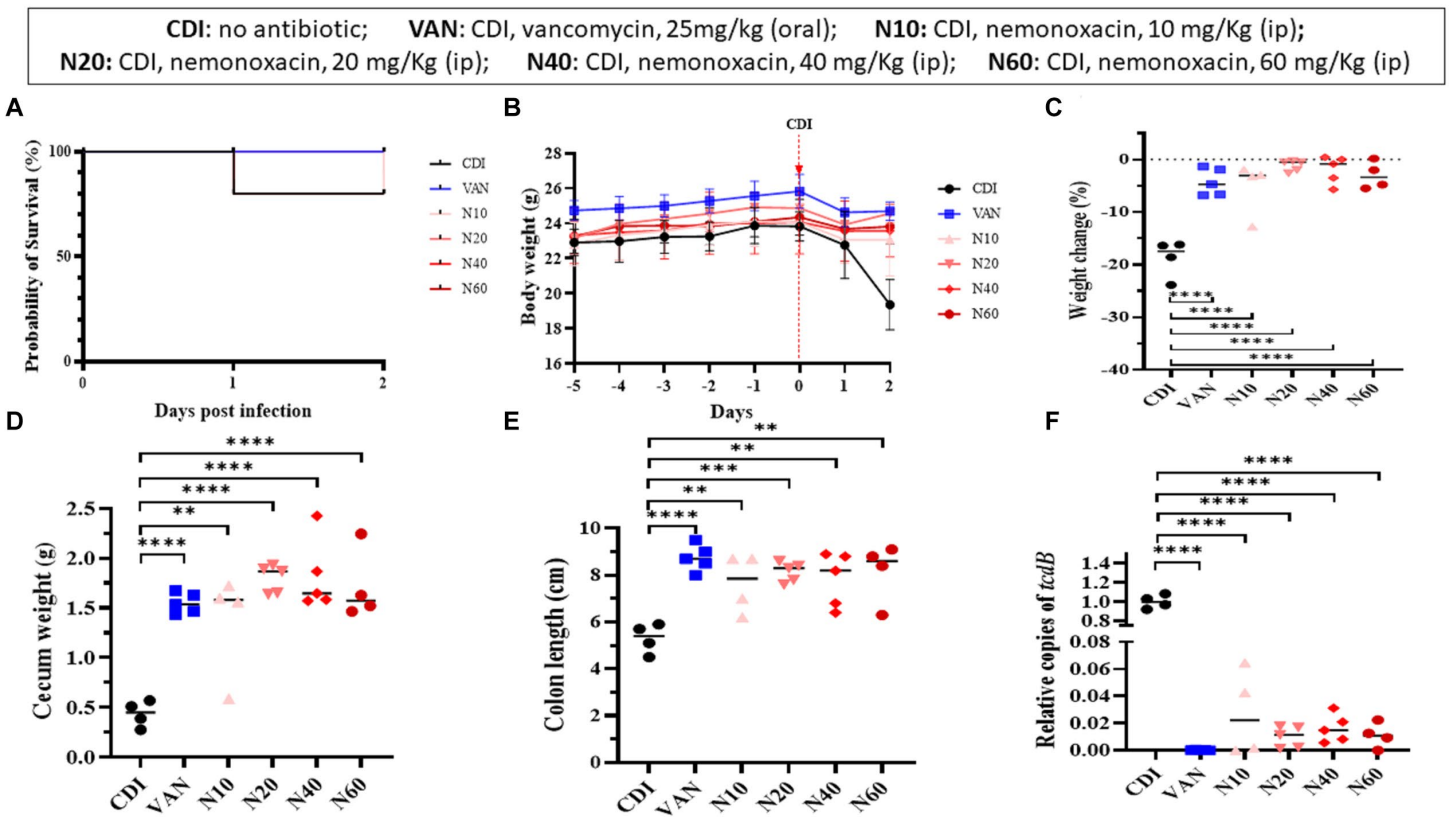
*In vivo* effects of various dosages (10, 20, 40, 60 mg/kg) of peritoneal nemonoxacin on inhibiting a clinical *C. difficile* isolate (105-NTUH 50) in a mouse model, compared with oral vancomycin (25 mg/kg). The effects were assessed through: **(A)** the proportion of survival after vancomycin or nemonoxacin therapy; **(B)** the alteration of body weight before and after *C. difficile* infection; the change of body weight **(C)**, cecum weight **(D)**, and colon length **(E)** after vancomycin or nemonoxacin therapy; and **(F)** relative *tcdB* level in stool after vancomycin or nemonoxacin therapy. Values are expressed as the mean ± standard deviation (*p < 0.05; **p < 0.01; ***p < 0.001; ****p < 0.0001 relative to control group). The absolute values of mice study and the mouse numbers in each replicate are detailed in Supplementary Table 1A.

In the mouse model infected with 105-NTUH 50, the survival rate of the 60 mg nemonoxacin treatment group and 80% for the untreated mice were 80% at 1 day after infection. At 2 days after infection, the survival rates were 80% for both the 10 and 60 mg nemonoxacin treatment groups (Figure 4A), resulting in a similar 2-day survival rate among mice treated with oral vancomycin (25 mg/kg), and mice treated with varying dosages of intraperitoneal nemonoxacin. The body weight of mice was detailed from the day of antibiotic cocktail treatment (day −5) to the day of sacrifice (day 2), as shown in Figure 4B. Mice that received 10 mg/kg (5.21% vs. 18.75%, *p* < 0.001), 20 mg/kg (1.2% vs. 18.75%, *p* < 0.0001), 40 mg/kg (1.93% vs. 18.75%, *p* < 0.0001), or 60 mg/kg (3.30% vs. 18.75%, *p* < 0.0001) of intraperitoneal nemonoxacin experienced significantly less weight loss than untreated mice (Figure 4C). Importantly, no significant difference in the changes of body weight was observed between vancomycin-treated mice and mice treated with any dose of nemonoxacin.

Mice that received 10 mg/kg (1.37 vs. 0.44 g, *p* < 0.01), 20 mg/kg (1.80 vs. 0.44 g, *p* < 0.00001), 40 mg/kg (1.81 vs. 0.44 g, *p* < 0.0001), or 60 mg/kg (1.71 vs. 0.44 g, *p* < 0.0001) of intraperitoneal nemonoxacin exhibited a higher cecum weight than untreated mice (Figure 4D). In addition, compared with untreated mice, mice receiving 10 mg/kg (5.30 vs. 7.65 cm, *p* < 0.01), 20 mg/kg (5.30 vs. 8.14 cm, *p* < 0.001), 40 mg/kg (5.30 vs. 7.82 cm, *p* < 0.01), or 60 mg/kg (5.30 vs. 8.15 cm, *p* < 0.01) of intraperitoneal nemonoxacin had a longer colon length (Figure 4E). Notably, no significant differences in the cecum weight or colon length were observed between vancomycin-treated mice and those treated with any dose of nemonoxacin.

To assess the relative *tcdB* level in mouse stool (Figure 4F), the relative copies of the *tcdB* gene in mice that received 10 mg/kg (0.02727 vs. 1.0018, *p* < 0.0001), 20 mg/kg (0.00996 vs. 1.0018, *p* < 0.0001), 40 mg/kg (0.01624 vs. 1.0018, *p* < 0.0001), or 60 mg/kg (0.01112 vs. 1.0018, *p* < 0.0001) of intraperitoneal nemonoxacin were significantly lower than that in untreated mice. Of importance, no significant difference was noticed in the relative copies of the *tcdB* gene between vancomycin-treated mice and mice treated with any dose of nemonoxacin.

### Combinative therapy of intraperitoneal nemonoxacin plus oral vancomycin in the mouse model

**Figure 5:**
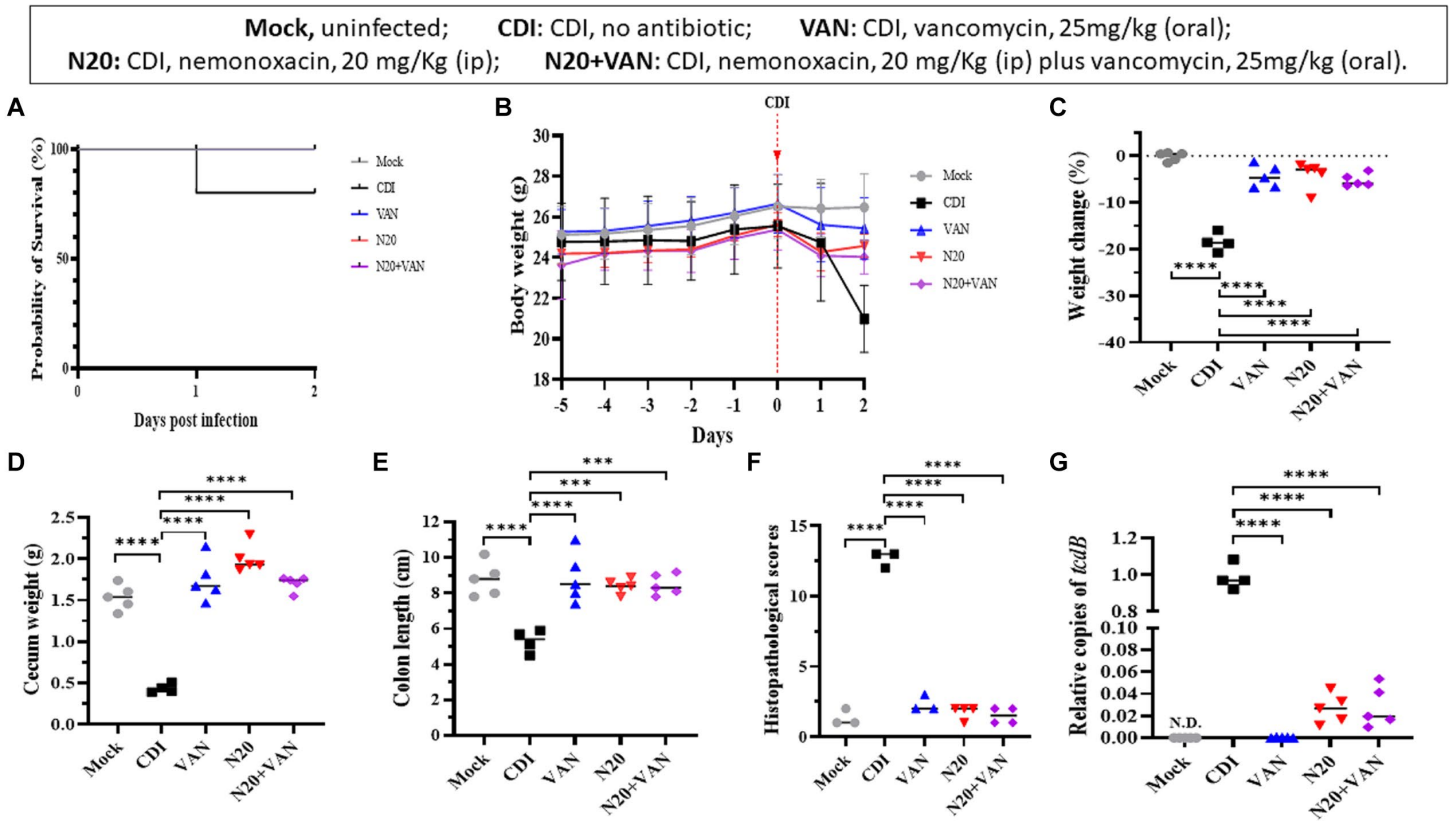
Comparisons of the *in vivo* effects of peritoneal nemonoxacin (20 mg/kg) monotherapy, oral vancomycin (20 mg/kg) monotherapy, and their combinative therapy on one clinical *C. difficile* isolate (105-NTUH 50), using a mouse model. The effects were assessed through: **(A)** the proportion of survival after vancomycin, nemonoxacin, or combinative therapy; **(B)** the alteration of body weight before and after *C. difficile* infection; the change of body weight **(C)**, cecum weight **(D)**, and colon length **(E)** after antibiotic therapy; **(F)** histopathological scores, and **(G)** relative *tcdB* level in stool after vancomycin or nemonoxacin therapy. Values are expressed as the mean ± the standard deviation (*p < 0.05; **p < 0.01; ***p < 0.001; ****p < 0.0001 relative to control group). The absolute values of mice study and the mouse numbers in each replicate are detailed in Supplementary Table 1B.

In the mouse model infected with 105-NTUH 50, the 2-day survival rate was 80% for the untreated group. In comparison, the mice treated with intraperitoneal nemonoxacin (20 mg/kg) and combinative therapy of oral vancomycin plus intraperitoneal nemonoxacin had a 100% survival rate (Figure 5A). The body weight of mice from day −5 to day 2 was recorded (Figure 5B). Compared with CDI mice treated with intraperitoneal nemonoxacin or oral vancomycin, those that received combinative therapy did not experience significantly less weight loss (Figure 5C). However, the cecum weight (Figure 5D), colon length (Figure 5E), histopathological scores (Figure 5F), and relative *tcdB* expression in stool (Figure 5G) among mice receiving combinative therapy were similar to CDI mice treated with intraperitoneal nemonoxacin or oral vancomycin. Moreover, histological sections of colon tissue from CDI mouse models treated with peritoneal nemonoxacin monotherapy, oral vancomycin monotherapy, and their combination therapy were proved in Supplementary Figure 2.

## Discussion

In the present study, we observed lower MICs of nemonoxacin against *C. difficile* compared with levofloxacin and ciprofloxacin, even in those harboring point mutations in the *GyrA* or *GyrB* genes. Similar to vancomycin, nemonoxacin failed to suppress spore outgrowth and germination in *C. difficile*. However, it effectively exhibited inhibitory effects against *C. difficile* in both the kinetic time-kill assay and the *ex vivo* co-culture model and these inhibitory abilities of nemonoxacin were consistent with those of vancomycin. Importantly, the ability of nemonoxacin in *in vitro* or *ex vivo* inhibiting *C. difficile* was consistent with vancomycin. In the mouse model, intraperitoneal injection of nemonoxacin, either alone or in combination with oral vancomycin, had a therapeutic effect in treating CDIs, which reduced body and cecum weight changes, alleviated bowel shortening, and decreased the expression of the *tcdB* gene, leading to a reduction in colon inflammation. Notably, the therapeutic efficacy between intraperitoneal nemonoxacin and oral vancomycin was similarly disclosed in the mouse model. Therefore, we believe that nemonoxacin has the potential to be administered parenterally in the treatment of patients with CDIs.

Nemonoxacin is an oral and intravenous quinolone that features a C-8-methoxy nonfluorinated structure and such modification leads to extended spectrum activity against clinically relevant pathogens (especially gram-positive bacteria), reduces mutant selection, and diminishes the incidence of toxic side effects (Li et al., 2010). Of importance, numerous *in vitro* studies have demonstrated lower MICs of nemonoxacin against clinical *C. difficile* isolates than those of commercially available fluoroquinolones (Lin et al., 2011; Liao et al., 2012). Similarly, regardless of whether clinical isolates or laboratory strains were tested, lower MICs of nemonoxacin compared with levofloxacin and ciprofloxacin were demonstrated in the present study. Moreover, for *C. difficile* harboring point mutations on *GyrA* or *GyrB* of QRDRs that lead to reduced susceptibilities to nemonoxacin, their MICs were still lower than those of other fluoroquinolones. On the other hand, the fecal excretion rate between intravenous metronidazole (ranging from 6 to 15%) (Wilcox and Howe, 1995) and nemonoxacin (approximately 6.1%) (TaiGenBiotechnology, 2023) was similar in previous reports. Given the relatively poor efficacies of parenteral metronidazole among CDI patients who are unable to take medications orally, these unique advantages of nemonoxacin were the leading reasons why we chose this non-fluorinated quinolone as a potential candidate to replace intravenous metronidazole in the treatment of individuals experiencing CDIs.

According to the IDSA guideline (McDonald et al., 2018), oral administration of vancomycin is the primary treatment for adults experiencing initial or severe episodes of CDIs. Therefore, in our study, oral vancomycin was chosen as the comparator to assess the effectiveness of nemonoxacin, rather than parenteral metronidazole. We reasonably believe that the effect of intraperitoneal nemonoxacin in inhibiting outgrowth and germination of *C. difficile* spores is similar to that of oral vancomycin, as only chemicals like hydrogen peroxide, chlorine dioxide, and various concentrations of hypochlorite have been evidenced for *in vitro* efficacies against *C. difficile* spores and are suitable for environmental disinfections (Barbut, 2015). Importantly, similar effectiveness between oral vancomycin and intraperitoneal nemonoxacin on study outcomes was disclosed in our *in vitro*, *ex vivo* co-culture, and mouse models. Clinically, nemonoxacin might be another anti-*C. difficile* agent that can be administered parenterally.

This study possesses several limitations. First, although one clinical isolate and another laboratory strain both with the lowest MIC of nemonoxacin and vancomycin were tested in the present study, the genetic linkage of theses *C. difficile* isolates with worldwide and Taiwan strains, as recognized by core-genome multilocus sequence typing and whole-genome comparative analysis, was limited. Therefore, an external validation of our finding on other isolate/stain is necessary. Second, because a two-day period of vancomycin or nemonoxacin therapy was adopted, the short-term therapeutic efficacy was only observed during the acute phase of CDIs in the present study. Third, no significant differences in study outcomes were observed between vancomycin-treated mice and those treated with any dose of nemonoxacin in the mouse model. However, for combinative study, we only chose a nemonoxacin dosage of 20 mg/kg and experiments detailing lower dosages of nemonoxacin were not conducted. Finally, our findings, despite being repeated four times, were derived from a limited number of mice and may not be sufficiently reliable. Therefore, further investigation is needed to assess the long-term efficacy of nemonoxacin in animal studies, including the potential development of quinolone resistance, and the effectiveness of combinative therapy of low dosage nemonoxacin. Additionally, a randomized controlled trial to compare the effectiveness of oral vancomycin and intraperitoneal nemonoxacin in treating patients with CDIs is necessary.

## Conclusion

Nemonoxacin similarly demonstrated effective inhibitory effects against *C. difficile* in both *in vitro* and *ex vivo* studies, compared with vancomycin. In the mouse model, intraperitoneal injection of nemonoxacin, whether administered alone or in combination with oral vancomycin, showed therapeutic efficacies in treating CDIs. Although our *ex vivo* experiments and animal studies indicate the potential role of nemonoxacin administered parenterally in the treatment of CDIs, more clinical investigations and laboratory work are warranted to validate our findings in human infections.

## Data Availability

The raw data supporting the conclusions of this article will be made available by the authors, without undue reservation.
