# Supplementary material for: Potential effectiveness of parenteral nemonoxacin in the treatment of Clostridioides difficile infections: in vitro, ex vivo, and mouse studies
Source: Front Microbiol. 2024 Aug 20;15:1418817. doi: 10.3389/fmicb.2024.1418817 (PMC11368742; doi:10.3389/fmicb.2024.1418817)
Supplement: Supplementary file 1 [file Data_Sheet_1.docx]

**Supplemental Figure 1.** Susceptibilities of 12 *C. difficile* bacteria to nemonoxacin, levofloxacin, and moxifloxacin.


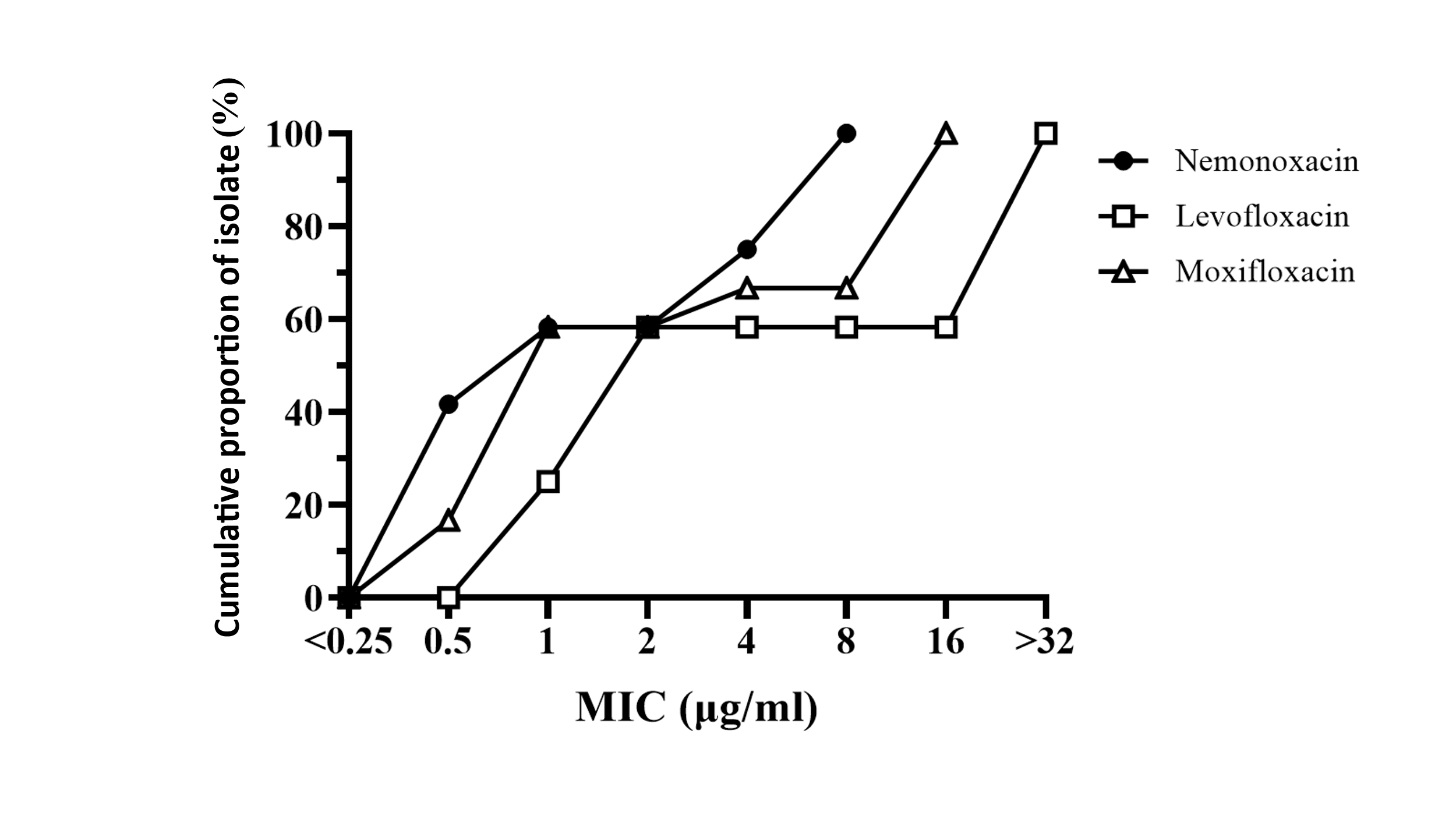


**Supplemental Figure 2.** Histological sections of colon tissue from CDI mouse models infected with a clinical isolate (105-NTUH 50) and treated with peritoneal nemonoxacin (20 mg/kg) monotherapy, oral vancomycin (20 mg/kg) monotherapy, and their combination therapy#.


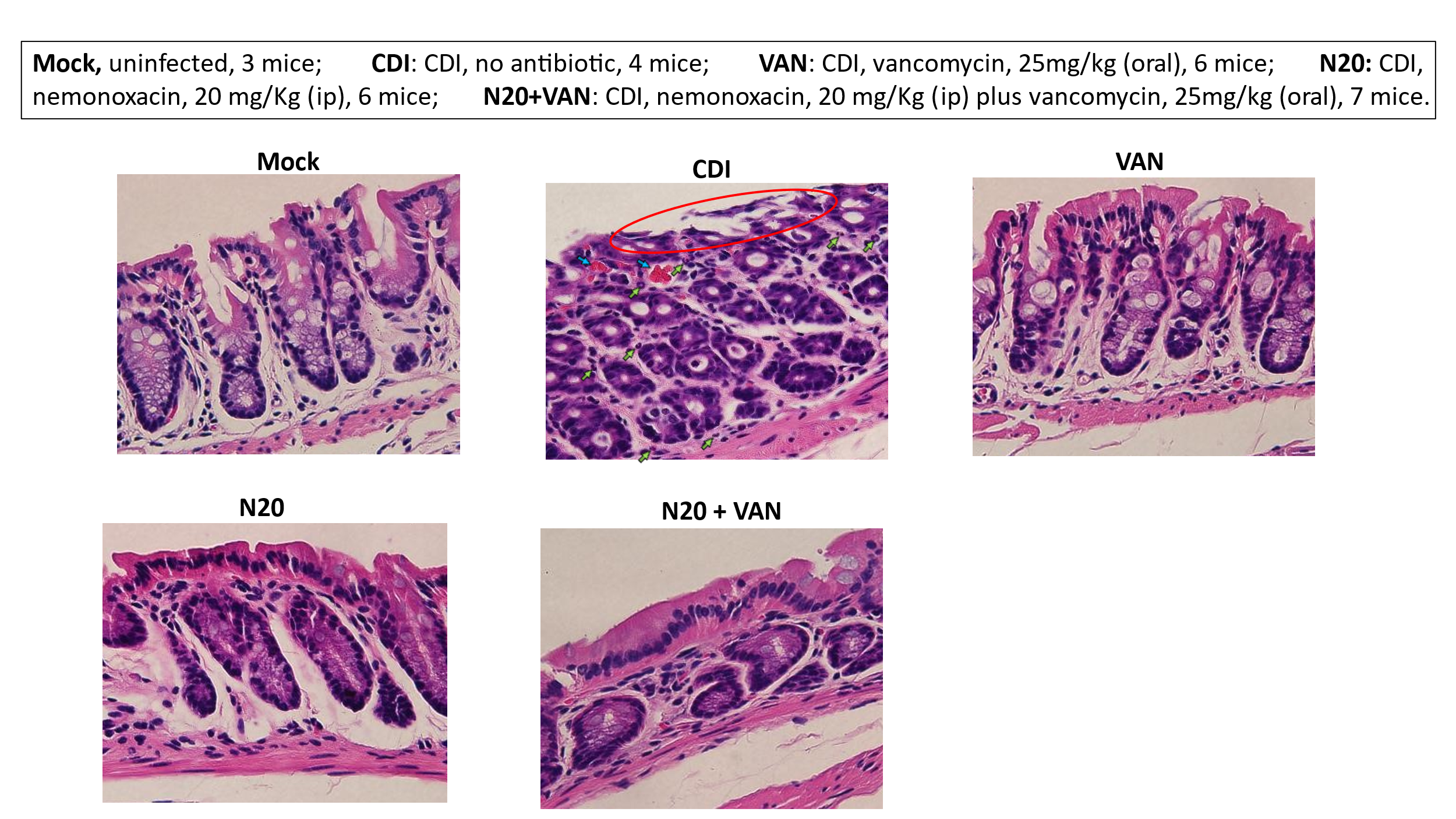


^#^ Seven-week-old male C57BL/6JNral mice were used. The number of tested mice is listed in the figure.

CDI = *Clostridioides difficil*e infection

**Supplemental Table 1.** The mice number and absolute value of mice study in each replicate: (A) various dosages (10, 20, 40, 60 mg/kg) of peritoneal nemonoxacin therapy (for Figure 4) and (B) nemonoxacin (20 mg/kg) monotherapy, oral vancomycin (20 mg/kg) monotherapy, and their combinative therapy (for Figure 5).

(A)

| Groups | Replicates | % (Mouse number) |  | Mean + standard deviation (Mouse number) | | | | |
| --- | --- | --- | --- | --- | --- | --- | --- | --- |
|  |  | Survival rate |  | Body weight (g) | Weight change (%) | Cecum weight (g) | Colon length, (cm) | *tcdB* copies |
| *C. difficile* infection | 1 | 80 (5) |  | $22.50\pm1.84$ (4) | $-14.42\pm3.11$ (4) | $0.45\pm0.15$ (4) | $6.28\pm0.65$ (4) | $0.9726\pm0.0514$ (4) |
|  | 2 | 100 (5) |  | $22.81\pm0.77$ (5) | $-14.30\pm3.11$ (5) | $0.53\pm0.15$ (5) | $5.50\pm0.48$ (5) | $0.9605\pm0.0477$ (5) |
|  | 3 | 80 (5) |  | $22.66\pm0.80$ (4) | $-14.70\pm3.70$ (4) | $0.41\pm0.14$ (4) | $6.23\pm0.83$ (4) | $0.9695\pm0.0579$ (4) |
|  | 4 | 80 (5) |  | $19.35\pm1.24$ (4) | $-18.75\pm3.09$ (4) | $0.44\pm0.11$ (4) | $5.30\pm0.55$ (4) | $1.0019\pm0.06121$ (4) |
| Vancomycin | 1 | 100 (5) |  | $24.06\pm0.78$ (5) | $-3.88\pm1.84$ (5) | $1.53\pm0.12$ (5) | $8.26\pm0.52$ (5) | $0.0006\pm0.0002$ (5) |
|  | 2 | 100 (5) |  | $24.38\pm0.68$ (5) | $-4.81\pm1.18$ (5) | $1.55\pm0.17$ (5) | $8.7\pm0.47$ (5) | $0.0005\pm0.0002$ (5) |
|  | 3 | 100 (5) |  | $24.15\pm0.84$ (5) | $-5.17\pm1.37$ (5) | $1.57\pm0.14$ (5) | $8.32\pm0.53$ (5) | $0.0003\pm0.0001$ (5) |
|  | 4 | 100 (5) |  | $24.70\pm0.47$ (5) | $-4.30\pm2.30$ (5) | $1.55\pm0.09$ (5) | $8.74\pm0.50$ (5) | $0.0003\pm0.0003$ (5) |
| Nemonoxacin 10 mg/ kg | 1 | 100 (5) |  | $23.08\pm0.88$ (5) | $-6.19\pm3.85$ (5) | $1.31\pm0.40$ (5) | $7.38\pm0.50$ (5) | $0.0328\pm0.0214$ (5) |
|  | 2 | 80 (5) |  | $22.63\pm0.81$ (4) | $-6.56\pm2.47$ (4) | $1.33\pm0.31$ (4) | $7.15\pm0.54$ (4) | $0.0363\pm0.0154$ (4) |
|  | 3 | 100 (5) |  | $23.71\pm1.00$ (5) | $-5.39\pm2.70$ (5) | $1.26\pm0.31$ (5) | $7.16\pm0.67$ (5) | $0.0359\pm0.0165$ (5) |
|  | 4 | 80 (5) |  | $23.06\pm1.79$ (4) | $-5.21\pm4.39$ (4) | $1.39\pm0.45$ (4) | $7.65\pm1.09$ (4) | $0.0273\pm0.0275$ (4) |
| Nemonoxacin 20 mg/ kg | 1 | 100 (5) |  | $25.04\pm1.07$ (5) | $-1.77\pm0.98$ (5) | $1.78\pm0.12$ (5) | $8.06\pm0.31$ (5) | $0.0106\pm0.0034$ (5) |
|  | 2 | 100 (5) |  | $24.97\pm1.16$ (5) | $-2.10\pm0.89$ (5) | $1.72\pm0.19$ (5) | $8.14\pm0.36$ (5) | $0.0110\pm0.0065$ (5) |
|  | 3 | 100 (5) |  | $24.58\pm0.86$ (5) | $-1.70\pm1.07$ (5) | $1.62\pm0.12$ (5) | $8.24\pm0.48$ (5) | $0.01157\pm0.0024$ (5) |
|  | 4 | 100 (5) |  | $24.56\pm0.50$ (5) | $-1.204\pm0.94$ (5) | $1.80\pm0.12$ (5) | $8.14\pm0.38$ (5) | $0.0100\pm0.0$070 (5) |
| Nemonoxacin 40 mg/ kg | 1 | 100 (5) |  | $24.69\pm0.94$ (5) | $-2.04\pm0.97$ (5) | $1.76\pm0.20$ (5) | $8.24\pm0.36$ (5) | $0.0171\pm0.0135$ (5) |
|  | 2 | 100 (5) |  | $24.83\pm0.95$ (5) | $-2.17\pm1.37$ (5) | $1.88\pm0.26$ (5) | $8.08\pm0.41$ (5) | $0.0155\pm0.0118$ (5) |
|  | 3 | 100 (5) |  | $24.33\pm1.19$ (5) | $-1.68\pm1.16$ (5) | $1.70\pm0.25$ (5) | $8.00\pm0.46$ (5) | $0.0117\pm0.0081$ (5) |
|  | 4 | 100 (5) |  | $23.57\pm1.34$ (5) | $-1.93\pm2.33$ (5) | $1.82\pm0.32$ (5) | $7.82\pm1.03$ (5) | $0.0162\pm0.0092$ (5) |
| Nemonoxacin 60 mg/ kg | 1 | 100 (5) |  | $24.79\pm0.91$ (5) | $-3.18\pm1.49$ (5) | $1.63\pm0.22$ (5) | $8.04\pm0.30$ (5) | $0.0168\pm0.0074$ (5) |
|  | 2 | 80 (5) |  | $23.89\pm0.46$ (4) | $-4.12\pm1.28$ (4) | $1.64\pm0.18$ (4) | $8.05\pm0.40$ (4) | $0.0138\pm0.0106$ (4) |
|  | 3 | 100 (5) |  | $24.75\pm1.15$ (5) | $-3.96\pm2.10$ (5) | $1.77\pm0.21$ (5) | $8.10\pm0.60$ (5) | $0.0202\pm0.0083$ (5) |
|  | 4 | 80 (5) |  | $23.82\pm0.87$ (4) | $-3.03\pm2.26$ (4) | $1.71\pm0.31$ (4) | $8.15\pm1.10$ (4) | $0.0111\pm0.0080$ (4) |

(B)

| Groups | Replicates | % (Mouse number) |  | Mean + standard deviation (Mouse number) | | | | |
| --- | --- | --- | --- | --- | --- | --- | --- | --- |
|  |  | Survival rate |  | Body weight (g) | Weight change (%) | Cecum weight (g) | Colon length, (cm) | *tcdB* copies |
| Mock | 1 | 100 (5) |  | $24.34\pm0.82$ (5) | $0.11\pm1.06$ (5) | $1.45\pm0.14$ (5) | $8.00\pm0.61$ (5) | Non-detected (5) |
|  | 2 | 100 (5) |  | $25.62\pm0.93$ (5) | $0.12\pm0.60$ (5) | $1.41\pm0.14$ (5) | $8.22\pm0.68$ (5) | Non-detected (5) |
|  | 3 | 100 (5) |  | $264.76\pm1.27$ (5) | $0.10\pm1.09$ (5) | $1.48\pm0.17$ (5) | $8.36\pm0.67$ (5) | Non-detected (5) |
|  | 4 | 100 (5) |  | $26.47\pm1.47$ (5) | $-0.17\pm0.84$ (5) | $1.53\pm0.14$ (5) | $8.78\pm0.86$ (5) | Non-detected (5) |
| *C. difficile* infection | 1 | 80 (5) |  | $22.41\pm1.10$ (4) | $-16.16\pm2.56$ (4) | $0.50\pm0.05$ (4) | $6.03\pm0.31$ (4) | $0.9777\pm0.0422$ (4) |
|  | 2 | 100 (5) |  | $24.30\pm1.03$ (5) | $-13.54\pm2.65$ (5) | $0.58\pm0.14$ (5) | $6.34\pm0.50$ (5) | $0.9839\pm0.0411$ (5) |
|  | 3 | 80 (5) |  | $21.93\pm1.44$ (4) | $-13.37\pm2.47$ (4) | $0.51\pm0.08$ (4) | $6.55\pm0.43$ (4) | $0.9887\pm0.0499$ (4) |
|  | 4 | 80 (5) |  | $20.99\pm1.43$ (4) | $-18.33\pm1.70$ (4) | $0.43\pm0.05$ (4) | $5.40\pm0.54$ (4) | $0.9863\pm0.0595$ (4) |
| Vancomycin | 1 | 100 (5) |  | $24.78\pm1.53$ (5) | $-4.11\pm2.32$ (5) | $1.69\pm0.25$ (5) | $8.52\pm0.89$ (5) | $0.0004\pm0.0003$ (5) |
|  | 2 | 100 (5) |  | $25.29\pm0.80$ (5) | $-5.19\pm1.51$ (5) | $1.66\pm0.16$ (5) | $8.48\pm0.53$ (5) | $0.0004\pm0.0001$ (5) |
|  | 3 | 100 (5) |  | $24.94\pm1.14$ (5) | $-5.94\pm1.09$ (5) | $1.52\pm0.10$ (5) | $8.28\pm0.38$ (5) | $0.0004\pm0.0002$ (5) |
|  | 4 | 100 (5) |  | $25.45\pm1.36$ (5) | $-4.472\pm2.14$ (5) | $1.74\pm0.24$ (5) | $8.88\pm1.26$ (5) | $0.0004\pm0.0002$ (5) |
| Nemonoxacin 20 mg/ kg | 1 | 100 (5) |  | $24.55\pm0.91$ (5) | $-4.79\pm2.20$ (5) | $1.72\pm0.28$ (5) | $8.36\pm0.49$ (5) | $0.0375\pm0.0133$ (5) |
|  | 2 | 100 (5) |  | $25.13\pm0.99$ (5) | $-3.74\pm1.61$ (5) | $1.80\pm0.26$ (5) | $7.78\pm0.66$ (5) | $0.0312\pm0.0120$ (5) |
|  | 3 | 100 (5) |  | $24.33\pm0.76$ (5) | $-3.90\pm1.53$ (5) | $1.73\pm0.20$ (5) | $7.74\pm0.53$ (5) | $0.0334\pm0.0128$ (5) |
|  | 4 | 100 (5) |  | $24.57\pm0.51$ (5) | $-4.08\pm2.55$ (5) | $2.00\pm0.15$ (5) | $8.40\pm0.36$ (5) | $0.0267\pm0.0119$ (5) |
| Nemonoxacin 20 mg/ kg+ vancomycin | 1 | 100 (5) |  | $24.25\pm1.04$ (5) | $-5.36\pm1.71$ (5) | $1.60\pm0.17$ (5) | $8.16\pm0.49$ (5) | $0.0314\pm0.0126$ (5) |
|  | 2 | 100 (5) |  | $24.58\pm0.78$ (5) | $-3.30\pm1.53$ (5) | $1.84\pm0.21$ (5) | $8.06\pm0.60$ (5) | $0.0319\pm0.0118$ (5) |
|  | 3 | 100 (5) |  | $24.37\pm0.56$ (5) | $-4.72\pm1.30$ (5) | $1.70\pm0.10$ (5) | $7.92\pm0.57$ (5) | $0.0329\pm0.0115$ (5) |
|  | 4 | 100 (5) |  | $24.03\pm0.74$ (5) | $-5.27\pm1.21$ (5) | $1.70\pm0.08$ (5) | $8.48\pm0.53$ (5) | $0.0283\pm0.0165$ (5) |
